# Soldiers' perceived versus actual heat strain in a jungle environment

**DOI:** 10.1186/2046-7648-4-S1-A21

**Published:** 2015-09-14

**Authors:** Alison Fogarty, Andrew Hunt, Catriona A Burdon

**Affiliations:** 1 Land Division, Defence Science and Technology Organisation, Melbourne, Australia; 2 Centre for Human and Applied Physiology, School of Medicine, University of Wollongong, Wollongong, Australia

## Introduction

Soldiers are regularly required to work in hot environments whilst wearing protective body armour (BA). However, BA is impermeable and decreases the torso surface area available for evaporative heat losses [1]. Consequently, an elevation in body core temperature was observed with early versions of BA [2,3]. In recent years, the size (and surface area coverage) of BA has decreased and laboratory simulations have shown that this newer BA does not increase the physiological load to the same extent as previous systems [4]. Anecdotally, however, Australian soldiers continue to report feeling an increased thermal burden when wearing BA. Therefore, we investigated the disconnect between experience and laboratory trials of the thermal impact of wearing BA in a warm jungle environment.

## Methods

Thirty-one Australian soldiers undertook two activities (three days of patrolling and a section competition including a march, a battle run, an obstacle course and a bayonet assault course) wearing either BA and webbing (BAW) or webbing (W) only while undertaking jungle training. Although the groups were not matched due to operational constraints, there were no significant differences between the groups in anthropometric measures or aerobic capacity. Heart rate (HR) and body core temperature (T_c_) were measured using a physiological monitoring system. Perceived heat illness symptoms were measured using the Environmental Symptoms Questionnaire (ESQ; 22 statements) [5]. Environmental conditions were measured using a wet bulb globe thermometer (WBGT).

## Results

The WBGT was 24-25 °C and 20 °C for the section patrol days and section competition respectively. The physiological measures (HR and T_c_) were not significantly different between the BAW and W groups during both activities. The summed ESQ rating was not different between groups during the patrol days, however six individual statements were higher with the BAW group. In contrast, after the section competition both the sum of ratings and 7 statements were significantly higher in the BAW group.

## Discussion

Similar to laboratory simulations, BA (with reduced surface area) did not impose a greater thermal strain on soldiers in a warm jungle environment. Despite this finding, the ESQ indicates that the soldiers wearing BA perceived that they were under greater thermal strain.

## Conclusion

The findings of the present study suggest that, if operational needs require soldiers to wear BA in a jungle environment, there is not an increased risk of personnel becoming a heat casualty. However, soldiers perceive themselves to be more uncomfortable and thus may be less able to concentrate on the mission.
